# Self-medication among pregnant women attending antenatal clinic at Makongoro health centre in Mwanza, Tanzania: a challenge to health systems

**DOI:** 10.1186/s12884-017-1642-8

**Published:** 2018-01-08

**Authors:** Karol J. Marwa, Agnes Njalika, Deodatus Ruganuza, Deogratias Katabalo, Erasmus Kamugisha

**Affiliations:** 1 Department of Pharmacology, Catholic University of Health and Allied Sciences, Mwanza, Tanzania; 2 Department of Parasitology, Catholic University of Health and Allied Sciences, Mwanza, Tanzania; 3 School of Pharmacy, Catholic University of Health and Allied Sciences, Mwanza, Tanzania; 4 Department of Biochemistry, Catholic University of Health and Allied Sciences, Mwanza, Tanzania

**Keywords:** Self-medication, Pregnancy, Predictors, Antenatal clinic and Tanzania

## Abstract

**Background:**

Self-medication is a universal challenge that requires attention because of the potential threat not only to the pregnant women but also to unborn child. Data on self-medication practice and predictors among pregnant women is lacking in Tanzania. Information on the effects of this practice to the pregnant woman and the foetus globally is also scanty.

**Methods:**

This was a cross sectional study which was conducted using face to face interview with 372 pregnant women at Makongoro health centre. Semi-structured questionnaires were used. Data were analysed using STATA 13 (Statistical Corporation, College Station, Texas, US).

**Results:**

A total of 372 pregnant women participated in the study. The prevalence of self-medication among pregnant women was 172 (46.24%). There was a significant statistical association between self-medication and occupation (*P* value =0.01), gestation age (*P* < 0.01) and education (*P* < 0.01). Age, marital status and gravidity were not associated with self-medication (*P* = 0.809, *P* = 0.243 and *P* = 0.922) respectively. When bivariate logistic regression was performed, occupation and education were the only determining factors for self-medication. Pregnant women who were unemployed, doing business and house wife were most likely to practice self-medication than employed pregnant women (*P* = 0.03; OR = 2.33; 95% CI, 1.06–5.31, *P* = 0.01; OR = 2.31; CI 1.21–4.41, *P* = <0.01, OR = 2.73, 95% CI 0.52–2.43) respectively. Pregnant women with no formal education, incomplete primary education, primary education and secondary education were most likely to practice self-medication than pregnant women with college or university education (*P* < 0.01, OR = 6.37 95% CI 2.37–19.03, *P* < 0.01, OR = 6.58, 95% CI 2.36–18.25, *P* < 0.01, OR = 3.78, 95% CI 1.89–7.56, *P* < 0.01, OR = 2.59 95% CI = 1.30–5.17). The leading illness/symptoms which led to self-medication among pregnant women attending clinic were malaria 56 (32.56%, morning sickness 44 (25.55%) and headache 33(19.19%). Drugs commonly used in self-medication among pregnant women were ant malarial 42 (24.42%), antiemetics 59 (34.30%) and analgesics 33 (19.19%).

**Conclusion:**

Prevalence of self-medication among pregnant women is high in Tanzania. This is a threat to the safety of the developing foetus and the pregnant woman. Therefore there is a need of interventions to minimize the practice among pregnant women.

**Electronic supplementary material:**

The online version of this article (10.1186/s12884-017-1642-8) contains supplementary material, which is available to authorized users.

## Background

Self-medication is defined as self-administration of medication not prescribed by or in a manner not directed by a physician [1] and is regarded as part of self-care among patients assuming the responsibility of the medical personnel to treat or prevent illnesses by using non-prescription or prescription only medicines (POMs) [2]. Self-medication may extend to prescription and herbal drugs and may be propagated by counselling or advice offered from health care professionals [2–4]. Self-medication carries serious risk of drug interactions, polypharmacy, misdiagnosis, excessive drug dosage use, prolonged drug use, incorrect drug choice, rare but severe adverse events, dependence or abuse and increased antimicrobial resistance [2, 5–8].

Self-medication is common in African countries including Tanzania [9–14]. Many studies have addressed the use drugs after prescriptions/directions from physicians; however data on self-medication during pregnancy is scanty.

Herbal drugs are commonly used in self-medication in developing countries [13–15]. The wide use of herbal drugs in pregnancy is also observed in developed countries [15] Norway being an example (36%) [16]. Studies have shown that herbal drugs are also not safe during pregnancy unlike what most people think [17] though information on effects to the foetus is limited [15].

The main reason for self-medication as reported from different countries include; the feeling that the condition /disease is mild thus not requiring doctor’s consultation, previous good experience of treating similar illness, prompted by a pharmacist, feeling of independence to take care of him/herself and non-availability of doctors [4, 14, 18–20]. Advertisement by pharmaceutical companies or their agencies on drugs has also been established as a promoting factor for self-medication [21].

Despite the fact that the use of prescription and non-prescription drugs is common among pregnant women [22, 23], most drugs used in clinical practice have limited information on the safety in pregnancy, hence not recommended for use in pregnant women [24, 25]. This is due to the fact that pregnant women are excluded from clinical trials owing to the fear of harming the mother or the developing foetus [26, 27]. The only available information on drug effects during pregnancy are derived from pre-clinical studies which involve animals and cannot be directly extrapolated to human beings including pregnant women [25, 27, 28], however such studies give us insight on the possibility of toxic effects to the foetus and the pregnant woman [27, 29].

Drug use cannot be avoided in pregnancy in acute illnesses which can harm the mother or foetus if left untreated [24]. In such situation drug use in these patients is based on risk-benefit ratio assessment whereby the potential benefits to the mother must outweigh the risks to the foetus [27]. Drugs effects in pregnancy can be detected in post marketing surveillance studies when a drug is introduced to a large population, nevertheless there is limited information on drug risk to the foetus and pregnant woman, consequently health professionals are deprived of this important information during prescribing and dispensing [27, 30].

There is Food and Drug Authority (FDA) classification placing drugs in categories in relation to safety in pregnancy from class A (the safest) to class X (the teratogenic group) [27]. Unfortunately only few drugs (40%) are listed in this classification [27] indicating that only a safety of few drugs can be predicted during pregnancy considering facts that self-medication is a common practice among pregnant women [31, 32] and presence of a large number of counterfeit drugs in developing countries and the world at large [33–35]. The illicit trade on counterfeit drugs makes self-medication dangerous to the pregnant women and the foetus in developing countries like Tanzania.

There has been expansion of primary health care facilities which include health centres and dispensaries over the past decades [36] in Tanzania. However lack and absenteeism of trained staff, unreliable drug and medical equipment supply chains, resource constrains and sometimes health system failure [37–39] affect delivery of antenatal care services to pregnant women in Tanzania thus driving this group to self-medication and alternative medicine seeking.

It is important to evaluate self-medication among pregnant women considering the above barriers in provision of antenatal care services to this population in Tanzania and a high prevalence of congenital anomalies among infants (29%) reported during birth in Mwanza, Tanzania with central nervous system as the most affected organ system [40]. Close monitoring of drug use during pregnancy is of paramount importance towards protecting the pregnant woman and the unborn foetus.

Therefore in this study we estimated the prevalence of self-medication and evaluated predictors of self-medication among pregnant women at Makongoro antenatal clinic in Mwanza, Tanzania.

## Methods

### Study area and subjects

This was a cross sectional study conducted at Makongoro antenatal clinic which is the largest antenatal clinic in Mwanza city in North West, Tanzania. The city has approximately half a million inhabitants, and is located on the southern shores of Lake Victoria [41]. Approximately more than 50 pregnant women attend at Makongoro clinic in a day. About 17,431 pregnant women seek health services at this clinic per year [42]. There are no admissions at the clinic, patients are referred to the nearby by Sekou-toure regional hospital. The clinic was chosen because it gives a representation of the entire population of the city and is located in the city centre.

### Sampling procedure

A prevalence of self-medication of 36% among pregnant women on the basis of previous data was used to calculate a sample size of 372 respondents using the Kish Leslie (1965) formula. A systematic sampling method was employed where by numbers were assigned on the daily patient register from 1 to 10; then all 1st, 3rd and 7th numbers were picked randomly. Pregnant women who were represented by these numbers were included in the study regardless of their gestation age until when the sample size was reached. The picked women were interviewed on the same date of their visit to the clinic.

### Data collection

A questionnaire was developed in English and translated into the local language (Swahili) and then administered through face to face interviews after consent from the respondents. Pre-testing of the questionnaire was done at the same clinic two weeks before to assess practicability and respondent’s understanding of the questionnaire. The questionnaire captured the information on the socio-demographic characteristics, self-medication and predictors for the practice. Antenatal care follow up cards were also reviewed to document socio-demographic characteristics and substantiate some of the information given during the interview. The questionnaire instrument can be found in Additional file 1.

### Statistical analysis

Data were analysed using STATA 13 (Statistical Corporation, College Station, TX, US). Chi-square and Fischer’s exact tests were performed for determining association between categorical variables where appropriate. Demographic and socio-economic variables were assessed using logistic regression. The potential associations were first assessed at a bivariate level; then, factor with a *P*-value <0.2 were entered into the multivariate model. Age group term was entered as a priori into the multivariate model. Stepwise backward logistic regression was used to determine whether these variables were independent factors for self-medication during pregnancy; odds ratios (OR) and 95% confidence intervals (95% CI) were reported as a measure of effect. *P* value of ≤0.05 was considered as statistically significant.

## Results

### Demographic characteristics

Three hundred and seventy two (372) out of 376 pregnant women gave a consent to participate in the study, of whom 221(59.1%) and 139 (33.4%) were aged 18–27 and 28–37 years respectively while only 12(3.2%) were ≥38 years. Majority 254 (68.3%) of participants were married while 95(25.6%) were unmarried. One hundred and four (28.0%) were business women while 70 (18.8%) were employed, 41(11%) unemployed and 157(42.1%) were house wives. Twenty (5.4%) had never attended any formal education, 38 (10.2%) did not complete primary education, 122(32.8%) had primary school education, 130(35.0%) had secondary education and 62(16.6%) were college/university graduates. All pregnant women (372) who consented completed the interview successfully. This could be due to the design of the interview which was short and pregnant women were interviewed just before seeing the doctor early in the morning whereby they were not tired and were still on the wait list.

### Self-medication

Self-medication during pregnancy was practiced by 172 (46.24%) pregnant women. Self-medication was 59 (57.84%) during first trimester (<12 weeks), 87 (49.43%) during second trimester (13–24 weeks) and 26 (27.66%) during third trimester (Table 1).

**Table 1:** Prevalence and factors associated with self-medication among pregnant women attending at Makongoro clinic

Variable	Yes	No	Chi square	*P* value
Age				
18–27	104 (47.06%)	117 (52.94%)	0.9694	0.809
28–37	63 (45.32%)	76 (54.68%)		
38–47	5 (45.45%)	6 (54.55%)		
> 47	0 (0.00%)	1 (100%)		
Marital Status				
Not Married	47 (49.47%)	48 (50.53%)	5.47	0.243
Married	111 (43.70%)	143 (56.30%)		
Separated	6 (50.00%)	6 (50.00%)		
Divorced	5 (62.50%)	3 (37.50%)		
Widowed	3 (100.00%)	0 (0.00%)		
Occupation				
Employed	20 (28.57%)	50 (71.43%)	11.3	0.01
Business	50 (48.08%)	54 (51.92%)		
Unemployed	20 (48.78%)	21 (51.22%)		
House wife	82 (52.23%)	75 (47.77%)		
Education				
No formal education	13 (65.00%)	7 (35.00%)	25.06	<0.01
Incomplete Primary school	25 (65.79%)	13 (34.21%)		
Primary School	64 (52.46%)	58 (47.54%)		
Secondary School	56 (43.08%)	74 (56.92%)		
College or University Level	14 (22.58%)	48 (77.42%)		
Gestation Age				
First Trimester (<12 weeks)	59 (57.84%)	43 (42.16%)	19.3	<0.01
Second Trimester (13–24 weeks)	87 (49.43%)	89 (50.57%)		
Third Trimester (>25 weeks)	26 (27.66%)	68 (72.34%)		
Gravidity				
1	55 (45.83%)	65 (54.17%)	1.9786	0.922
2	51 (43.97%)	65 (56.03%)		
3	41 (46.59%)	47 (53.41%)E		
4	15 (50.00%)	15 (50.00%)		
5	5 (50.00%)	5 (50.00%)		
6	4 (57.14%)	6 (42.86%)		
7	1 (100.0%)	0 (0.00%)		

There was a significant statistical association between self-medication and occupation (*P* value =0.01), gestation age (*P* < 0.01) and education (P < 0.01). Nevertheless age, marital status and gravidity were not associated with self-medication (*P* = 0.809, *P* = 0.243 and *P* = 0.922) respectively (Table 1).

When bivariate logistic regression was performed, only occupation and education were the determining factors for self-medication (Table 2). Pregnant women who were unemployed 20 (48.78%), doing business 50(48.08%) and house wives 82 (52.23%) were most likely to self-medicate than employed pregnant women 20 (28.57%) (*P* = 0.03; OR = 2.33; 95% CI, 1.06–5.31, *P* = 0.01; OR = 2.31; CI 1.21–4.41, *P* = <0.01, OR = 2.73, 95% CI 0.52–2.43) respectively. Pregnant women with non-formal education 13 (65.00%), incomplete primary education 25 (65.79%), primary education 64 (52.46%) and secondary education 56 (43.08%) were most likely to practice self-medication than pregnant women with college or university education 14(22.58%) (*P* < 0.01, OR = 6.37 95% CI 2.37–19.03, *P* < 0.01, OR = 6.58, 95% CI 2.36–18.25, *P* < 0.01, OR = 3.78, 95% CI 1.89–7.56, *P* < 0.01, OR = 2.59 95% CI = 1.30–5.17).

**Table 2:** Predictors for self-medication among pregnant women attending clinic

Variable	N	*n* (%)	Odd Ratio	95% CI	*P* Value	adjusted Odd Ratio	95% CI	*P* Value
Age								
18–27	221	104 (47.06%)	1			1		
28–37	139	63 (45.32%)	0.93	0.6–142	0.74	1.04	0.66–1.64	0.88
38–47	11	5 (45.45%)	0.93	0.27–3.16	0.91	0.88	0.24–3.13	0.84
> 47	1	0 (0.00%)	1			1		
Occupation								
Employed	70	20 (28.57%)	1			1		
Business	104	50(48.08%)	2.31	1.21–4.41	0.01	1.38	0.67–2.83	0.38
Unemployed	41	20 (48.78%)	2.33	1.06–5.31	0.03	1.57	0.6–3.79	0.32
House wife	157	82 (52.23%)	2.73	1.49–5.00	<0.01	1.12	0.52–2.42	0.78
Education								
College or University	62	14 (22.58%)	1			1		
No formal education	20	13 (65.00%)	6.37	2.37–19.03	<0.01	7.52	2.15–26.28	0.02
Incomplete Primary	38	25 (65.79%)	6.59	2.69–16.15	<0.01	6.56	2.36–18.35	0.04
Primary School	122	64 (52.46%)	3.78	1.89–7.56	<0.01	3.64	1.59–8.32	0.02
Secondary School	130	56 (43.08%)	2.59	1.3–5.19	<0.01	2.50	1.2–5.22	0.01

Conversely when multivariate logistic regression was performed, education was the only factor established to be associated with self-medication (Table 2).

The leading illness/symptoms which necessitates self-medication among pregnant women attending clinic were malaria 56 (32.56%, morning sickness 44 (25.55%) and headache 33(19.19%) (Table 3).

**Table 3:** Common cause and illness/symptoms that necessitates self-medication among pregnant women

No.	Illness	Self-Medication
1	Malaria	56 (32.56%)
2	Urinary Tract Infection	16 (9.3%)
3	Morning Sickness	44 (25.55%)
4	Heartburn	4 (2.34%)
5	Headache	33 (19.19%)
6	Asthma	3 (1.74%)
7	Epilepsy	2 (1.16%)
8	Hypertension	2 (1.6%)
9	Cough & Cold	9 (5.25%)
10	Diarrhoea	1 (1.58%)
11	Helminth’s	1 (1.58%)
12	None	0 (0%)
13	Fungal Infection	1 (0.58%)
	TOTAL	172 (100%)

Drugs commonly used in self-medication among pregnant women were ant malaria 42(24.42%), antiemetic 59 (34.30%) and analgesics 33 (19.19%) (Table 4).

**Table 4:** Drugs commonly used in self-medication among pregnant women

No.	Illness	Self-Medication
1	Antimalarial	42 (24.42%)
2	Antibiotics	17 (9.58%)
3	Antiemetic	59 (34.30%)
4	Analgesics	33 (19.19%)
5	Antiasthma	3 (1.74%)
6	Antiepileptic	2 (1.16%)
7	Antihypertensive	2 (1.16%)
8	Cough & Cold Remedies	9 (5.23%)
9	Heartburn	3 (1.74%)
10	Antihelmintics	2 (1.16%)
	Total	172 (100%)

The overall use of traditional herbs among pregnant women was 94(25.3%). The use of traditional herbs among pregnant women who self-medicated with modern drugs was significantly higher 57(33.2%) than among pregnant women who did not self-medicate with modern drugs 37(18.5%) (*P*-value 0.001) as shown in Table 5.

**Table 5:** Self-medication with modern drugs and herbal drugs use among pregnant women

Self-medication	Herbal drugs use		Total	Chi2	*P*-value
	Yes	No			
Yes	57 (33.1%)	115 (66.9%)	172 (100%)	10.49	0.001
No	37 (18.5%)	163 (81.5%)	200 (100%)		
Total	94 (25.3%)	278 (74.7%)	372 (100%)		

## Discussion

The present study was carried out to establish prevalence and predictors of self-medication among pregnant women attending antenatal clinic in Mwanza, Tanzania. Prevalence of self-medication during pregnancy 172 (46.24%) was high. These findings are comparable to other developing countries particularly South Africa [31] and lower than Ethiopia(20.4%) [32].

This study has identified level of education as a strong predictor of self-medication among pregnant women (Tables 1 and 2). Pregnant women with non-formal education, incomplete primary education, primary education and secondary education were most likely to practice self-medication than pregnant women with college or university education. These findings are similar to those documented in developing countries like Sudan, Sri-Lanka, Mexico, Ghana and Nigeria [5, 20, 43–45] but different to developed European countries where higher levels of education is associated with self-medication [10, 18, 46]. The educated patients in developing countries may have a high income as most are employed therefore can afford health care service hence would not prefer self-medication. Low income is a predictor of self-medication in poor/developing countries [14, 45, 47] while high education level is associated with promptness in seeking care from health care providers [48] and attendance to clinic [49].

Conversely in developed countries people with high level of education opt for self-medication because there are internet pharmacies and these patients can easily understand drug leaflets hence they believe they can treat mild illnesses without consulting a doctor whereas the illiterate patients may not understand the drug leaflets and information on internet on drugs therefore relying on health care services which are reliable and affordable.

Occupation was also strong predictor for self-medication among pregnant women attending antenatal clinic (Table 1). Pregnant women who were unemployed, doing business and house wives were most likely to self-medicate than employed pregnant women. As shown above, high level of education was not associated with self-medication. Employment is associated with level of education. The recorded low level of self-medication among employed pregnant women could be attributed to their high level of education too which is the opposite with regards to unemployed or house wives. These findings are consistent with data from other developing countries like India [50].

Self-medication was highest during first trimester and decreased as gestation age increased where by less pregnant women self-medicated in the third trimester of pregnancy (Table 1). This finding is an alarmingly threat because drug exposure in this period is likely to cause congenital malformations [51, 52] considering high prevalence of congenital malformations reported during birth in our study area [40]. The higher self-medication practice in the first trimester may be attributed to the occurrence of many symptoms, discomforts and illness such as nausea or vomiting, headache, dizziness and fevers in the first trimester than other trimesters during pregnancy. However, when Multivariate logistic regression was performed, gestation did not emerge as a strong predictor for self-medication in pregnancy (Table 2).

Age was not observed as a predictor of self-medication similar to documentations in Nigeria [20] and different to findings in Portugal [53].

Self-medication with herbal drugs was substantially high (25.3%) among pregnant women. Surprisingly even those who did not self-medicate with modern drugs were also using herbal drugs though to a lesser extent than those who self-medicated with modern drugs as shown in Table 5. This is not surprising because self-medication with traditional herbal drugs is part of culture in Africa due to its availability, affordability and easy access [54]. Findings from this study are similar to other African countries particularly South Africa, where self-medication with herbal drugs during pregnancy is common [31]. The wide use of herbal drugs in pregnancy is also observed in developed countries Norway being an example (36%) [16, 55]. The high use of herbal drugs in developed and developing countries may be due to promotions and beliefs that herbs are natural and safe which attract pregnant women who are in most cases concerned about the health of their unborn children [55].

The prominent illness/symptoms which necessitate self-medication among pregnant women attending clinic were malaria, morning sickness and headache (Table 3). Malaria being among the leading illnesses among pregnant women is not surprising because women in malaria endemic areas like Mwanza are at a higher risk of being infected with malaria which is often associated with headache. Motion sickness is a common symptom experienced in pregnancy especially in the first trimester matching with the findings from the present study that most pregnant women self-medicate when they have nausea/vomiting.

Drugs commonly used in self-medication among pregnant women were antimalarial, antiemetics and analgesics. The use of drugs matches with prominent symptoms/illnesses compelling self-medication among pregnant women as discussed above. Self-medication with antimalaria drugs is already known to be a common practice among communities in malaria endemic countries like Tanzania. This is similar to the observed findings among pregnant women in this study [56–58]. Though malaria is endemic in Tanzania drug use is restricted for safety purposes in the first trimester (Artemisinin based combination therapies (ACTs) due to teratogenicity potential and third trimester (Quinine and its derivatives) due to ability to induce abortion and increased risk of hypoglycaemia as per WHO recommendation [59].

The antimalarial drugs mostly used in self-medication were Sulphadoxine-Pyrimethamine (54%) known as SP and Artemether-Lumefantrine (40%) known as ALU. The higher use of Sulphadoxine-Pyrimethamine (SP) which is used as drug of choice in Intermittent Preventive Therapy (IPT) among pregnant women and provided as DOT may be attributed to perception that the drug is used in treatment of malaria in pregnancy which is misleading since the drug is reserved for prevention of malaria infection only among pregnant women. Artemether-Lumefantrine is a first line drug for treatment of uncomplicated malaria in Tanzania but contraindicated in the first trimester of pregnancy [59] thus rising a concern on the safety of the unborn child resulting from self-medication.

## Study limitations

The current study did not evaluate the impact of self-medication to the pregnant women and infants during birth among those who practiced self-medication. Another limitation was that respondents were not asked if they reported their drug use to the nurses and doctors at the clinic. This information could help doctors in decision making during prescribing thus considering issues like drug interactions and overdose (repetition in using the same drug). It should also be borne in mind that the completeness of reporting of the past self-medication is prone to recall bias and the position for the first sample was chosen arbitrarily. Sensitivity analysis restricted to women on third trimester who have a full overview of the pregnancy was not done. Women in early pregnancy might have not have the chance yet to use medications consequently affecting the reported results on drugs commonly used in pregnancy.

This study has addressed a gap in knowledge on self-medication during pregnancy in the area. Findings from the present study is expected to raise awareness among health care providers especially clinicians and the ministry of health thus calling for appropriate intervention(s) to prevent unnecessary drug use and control self-medication among pregnant women .

### Conclusion

Prevalence of self-medication with modern drugs and herbal drugs among pregnant women in Tanzania is alarmingly high. The practice is common among illiterate, unemployed and during first trimester of pregnancy.

## Additional file

Additional file 1: Self-medication Questionnaire Responses. The data comprises the results of the questionnaires administered to 372 pregnant women attending antenatal clinic in Tanzania to assess their self-medication practice and predictors. (DOCX 18 kb)

## Abbreviations

ACTs: Artemisinin based combination therapies
ALU: Artemether-Lumefantrine
DOT: Directly Observed Therapy
FDA: Food and Drug Authority
IPT: Intermittent Preventive Therapy
POMs: Prescription Only Medicines
SP: Sulphadoxine-Pyrimethamine
WHO: World Health Organization
