# Supplementary material for: Self-medication among pregnant women attending antenatal clinic at Makongoro health centre in Mwanza, Tanzania: a challenge to health systems
Source: BMC Pregnancy Childbirth. 2018 Jan 8;18:16. doi: 10.1186/s12884-017-1642-8 (PMC5759229; doi:10.1186/s12884-017-1642-8)
Supplement: Additional file 1: — Self-medication Questionnaire Responses. The data comprises the results of the questionnaires administered to 372 pregnant women attending antenatal clinic in Tanzania to assess their self-medication practice and predictors. (DOCX 18 kb) [file 12884_2017_1642_MOESM1_ESM.docx]

**SECTION A; Socio- demographic data**

1. Age (years)………………………………………….

2. Marital status

1. Single ( )
2. Married ( )
3. Separated ( )
4. Divorced ( )
5. Widowed ( )

3. Occupation

1. Business ( )
2. Employed ( )
3. Unemployed ( )
4. Housewife ( )
5. Others ………………………

4. Highest level of education completed

1. No formal education ( )
2. Incomplete primary school ( )
3. Primary school ( )
4. Secondary school ( )
5. College or University level ( )

5. Residence

1. Rural ( )
2. Urban ( )

**SECTION B; Obstetric History**

6. Gravidity……………………………………………

7. Parity………………………………………………….

8. Gestation age…………………………………………

9. Mode of previously deliveries

1. Vaginal ( )
2. Assisted vacuum ( )
3. Caesarian. ( )

10. Previously bad obstetric history

1. Miscarriage ( )
2. Still born ( )
3. Premature ( )
4. Intra uterine fetal death ( )
5. None. ( )

11. Have you ever used any medication in this pregnancy?

1. Yes ( )
2. No ( )

12. If YES. How did you get these drugs?

1. Prescribed by the doctor ( )
2. Self ordered from the pharmacy ( )
3. Others specify………………………

13. Do you know these drugs may have side effects to the mother and child?

1. Yes ( )
2. No ( )

14. What were the conditions forced/made you to use these medication/drugs?

1. Malaria ( )
2. Urinary tract infection ( )
3. Morning sickness (nausea, vomiting) ( )
4. Heartburn ( )
5. Headache ( )
6. Asthma ( )
7. Epilepsy ( )
8. Hypertension ( )
9. Cough and colds ( )
10. Others………………………………………….

15. What kind of drugs do you usually use without a prescription?

1. Ant malaria ( )
2. Antibiotics ( )
3. Antiemetic ( )
4. Analgesics ( )
5. Antiasthma ( )
6. Ant epilepsy ( )
7. Antihypertensive ( )
8. Cough and cold remedies ( )
9. Others …………………………

Others specify…………………………

16. Which antimalarial agents do you use when you feel/confirm you have malaria?

a. SP

b. Artemether lumefantrine(ALU)

c. Quinine

d.Others .Mention …………………………………………………..

17. Do you normally check and read the accompanied leaflet content?

1. Yes ( )
2. No ( )

18. If YES; which information’s do you get from this leaflet?

1. …………………………………….
2. ………………………………………
3. … …………………………………………

19. If No; what made you not to read this leaflet?

1. I don’t know to read
2. I don’t know English
3. Drugs do not have leaflets.

20. Do you use herbal drugs during pregnancy?

1. Yes ( )
2. No ( )

21. If YES; in which conditions made you use these drugs?

1. …………………………………….
2. …………………………………….
3. …………………………………….

22. Do you smoke?

1. Yes ( )
2. No ( )

23. Do you take alcohol?

1. Yes ( )
2. No ( )
